# Management of Trochanteric Fractures: Are We NICE Compliant?

**DOI:** 10.7759/cureus.47038

**Published:** 2023-10-14

**Authors:** Rohit S Kumar, Effie Menyah, Azeem Thahir, Raman Thakur, Lindiwe Malindzisa, Jai Relwani

**Affiliations:** 1 Trauma and Orthopaedics, William Harvey Hospital, Ashford, GBR

**Keywords:** intramedullary nail, hip fracture, nice guidelines, extracapsular hip fracture, sliding hip screw, trochanteric fractures

## Abstract

Introduction: The National Institute for Health and Care Excellence (NICE) updated the 2011 hip fracture management guidelines on January 6, 2023, suggesting that clinicians offer sliding hip screws in preference to intramedullary nails for trochanteric fractures above and including the lesser trochanter except reverse oblique fractures. This study aims to assess the compliance of our hospital with the updated guidelines while comparing the results with our performance prior to the update together with the national average.

Materials and methods: A retrospective observational study was done to analyse if trochanteric fractures managed surgically were compliant with NICE guidelines. Pathological fractures secondary to tumours and AO/OTA 31A1.1 fractures were excluded. Fractures were classified using the 2018 AO/OTA classification system independently by two authors, with a review from a senior consultant if there was interobserver variation. Group A (n=60) included trochanteric fractures managed surgically three months prior to the update, while Group B (n=46) included patients managed operatively three months following the update.

Results: The compliance rates for Group A and Group B were similar at 88.33% and 89.13%, respectively, while the national average was about 67% over the course of six months.

Discussion: 31A2 fractures showed higher rates of non-compliance in both groups. Non-compliance was thought to be multifactorial: surgeon bias, inaccurate classification of fractures and a lack of awareness of guidelines.

Conclusions: While there is scope for improvement, district general hospitals can achieve high rates of compliance. Educating and training doctors could help improve compliance.

## Introduction

A hip fracture is a serious injury needing hospital admission, with 75,000 fractures reported each year in the United Kingdom [1]. In 2018, the National Hip Fracture Database (NHFD) reported that over one billion pounds were spent on the management of hip fractures [2]. By 2033, it is projected that the incidence of hip fractures could reach 100,000, costing the National Health Service £3.6-5.6 billion [3]. Hip fractures have a mortality rate of 7% within 30 days and 30% at one year [4].

To improve outcomes and quality of healthcare following hip fractures, the National Institute for Health and Care Excellence (NICE) in 2011 developed the first evidence-based guidelines in the United Kingdom for the management of hip fractures in individuals aged 18 years and older [5]. The guideline suggests that clinicians offer extramedullary fixation methods such as sliding hip screws (SHS) in preference to intramedullary nails (IMNs) for trochanteric fractures (TFs) above and including the lesser trochanter [5].

NICE updated the hip fracture management guidelines on January 6, 2023, to account for the change in AO/OTA fracture classification in 2018 [5,6]. The guideline suggested that SHS should be considered in preference to IMN in the management of 31A1 and A2 fractures, while IMN was recommended for A3 fractures [5]. These guidelines were developed after a systematic review of evidence that failed to show any benefit of IMN over SHS in 31A1 and A2 fractures [5].

Our study assessed our compliance with the recently updated NICE guidelines and compared the results with our performance prior to the update and the national performance.

## Materials and methods

A retrospective observational study was conducted in our district general hospital after obtaining approval from the institutional review board (ID no.: RN1189605). Information regarding the change in NICE guidelines for hip fracture management was disseminated during the monthly trauma and orthopaedic departmental clinical governance meeting prior to commencing the study. All extracapsular neck of femur fractures treated three months before and after the update were identified from the hospital hip fracture database. Only fractures managed by surgical intervention were included in the study. Pathological TFs due to tumours were excluded, as the management of these fractures follows different treatment principles. Isolated greater or lesser trochanter fractures (AO/OTA classification 31A1.1 fractures) were excluded. Demographic data including patient age and sex were collected.

Patients were divided into two cohorts: Group A included patients who underwent surgical fixation of TF over a period of three months prior to the update (October 6, 2022 to January 5, 2023), while Group B consisted of patients who were operated after the update, i.e., from January 6, 2023 to April 5, 2023. The study was conducted in April 2023, and hence, we collected data for a period of three months following the update and collected data for the same time period prior to the update to facilitate comparison. 

Antero-posterior and lateral radiographs of the fractured hips were reviewed independently by two authors and classified according to the 2018 AO/OTA classification [6]. According to the 2018 AO/OTA classification [6], 31A1 and A2 fractures had a fracture across the trochanters with a competent and incompetent lateral wall, respectively. A competent lateral wall implied that the lateral wall thickness was greater than 20.5 mm. Both 31A1 and A2 fractures were classified into subgroups based on fracture pattern and comminution. 31A3 fractures, also referred to as reverse oblique fractures, had a fracture running from the lesser trochanter distally. A senior consultant reviewed the radiographs to exclude interobserver variation in fracture classification. Fractures sustained by patients in Group A were classified using the updated system to facilitate comparison with Group B. The implant used in surgical fixation of fractures was assessed for compliance with the guidelines. In our institution, short IMNs are not used. Only fractures classified as 31A1.2, 31A1.3, 31A2.2 and 31A2.3 treated with an extramedullary implant (such as SHS) and 31A3.1, 31A3.2 and 31A3.3 fractures treated with an IMN were recorded as compliant with the updated guideline. 

## Results

A total of 107 patients presented with TFs during this time period. One patient was referred to a major trauma centre due to complex medical problems that required expertise in management and was excluded from the study. Of the 106 patients included, 60 were in Group A and 46 were in Group B. The average age was 82.37 years (range 51-103, standard deviation 9.92). The gender distribution was 31 males and 75 females. A breakdown of the age and sex distribution is detailed in Table 1.

**Table 1:** Age and sex distribution across groups.

	Male	Female	Mean age in years (range, standard deviation)
Group A (n=60)	15	45	82.63 (51-99, 9.67)
Group B (n=46)	16	30	82.02 (55-103, 10.33)

Group A had 88.33% (n=53) compliance with guidelines. Among the 21 patients treated with an IMN, 14 classified as 31A3 were treated as per guidelines, while one of 20 classified as A1 fracture and six of 26 classified as A2 were non-compliant and were treated with IMN instead of SHS.

The compliance rate of Group B was similar, at 89.13% (n=41). Group B consisted of 16 31A1 fractures, and all received NICE compliant surgery, i.e., they were treated with a SHS. Three of the 17 patients classified as 31A2 fractures had non-compliant surgery (IMN). The 31A3 fracture subgroup had 13 patients and two were managed with a non-compliant surgical procedure (SHS). A detailed classification of fractures and compliance across groups is enumerated in Table 2 [6].

**Table 2:** Fracture classification across groups as per 2018 AO/OTA classification versus compliance with NICE guideline for hip fractures. NICE: National Institute for Health and Care Excellence.

Fracture classification	Group A (n=60)		Group B (n=46)	
	Compliant	Non-compliant	Compliant	Non-compliant
A1.2	15	0	11	0
A1.3	4	1	5	0
A2.2	15	2	13	1
A2.3	5	4	1	2
A3.1	2	0	4	0
A3.2	2	0	0	0
A3.3	10	0	7	2

## Discussion

**Figure 1:**
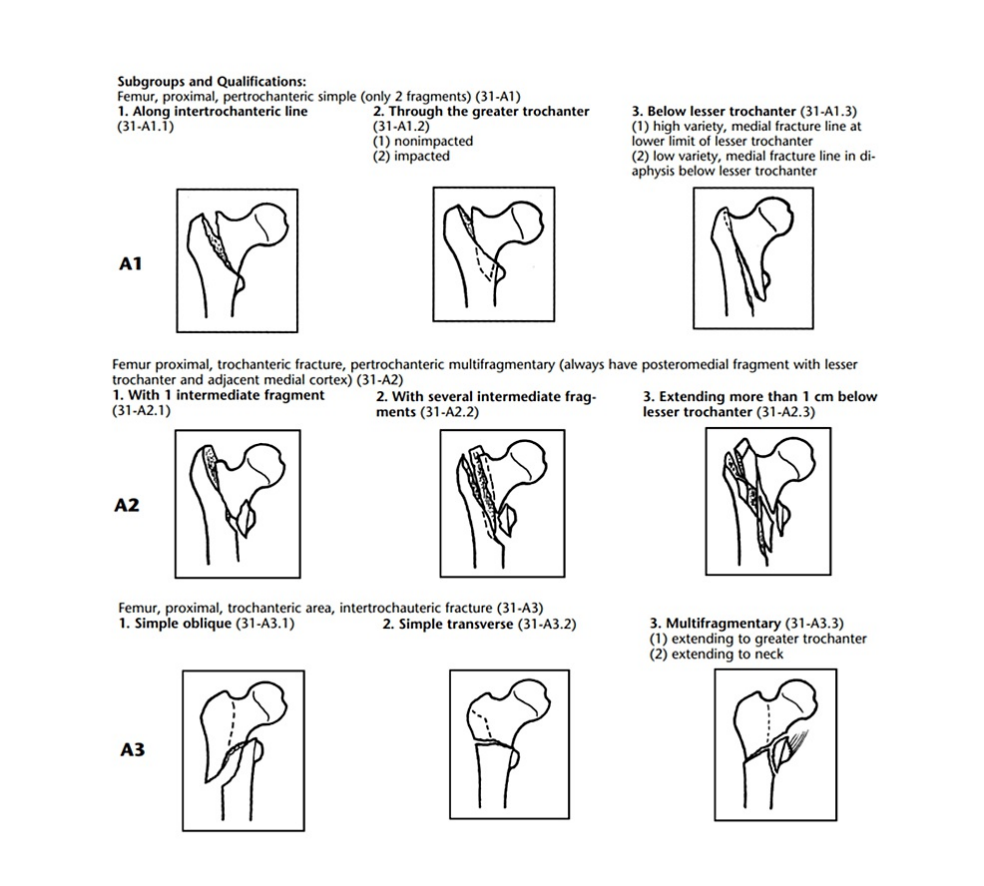
A1 fractures have a simple fracture line extending from the greater trochanter to the medial femoral cortex, while A2 fractures are multifragmentary pertrochanteric fractures and always have a posteromedial fragment. A3 fractures have a fracture line running from the lesser trochanter to the lateral femoral cortex distal to the lesser trochanter and are further classified based on fracture orientation and comminution. Source: Reproduced from Marsh et al. [8] with permission from the Journal of Orthopaedic Trauma.

The 2011 NICE guideline [5] for hip fracture management was based on the 1990 AO/OTA classification [7], which described the stability of a fracture based on the integrity of the posteromedial cortex. This classification divided TF into three broad groups based on fracture stability: 31 A1, A2 and A3, with each fracture further divided into further subgroups. While both 31A1 and A2 fractures had fracture lines running from the greater to the lesser trochanter, 31A1 fractures were simple, whereas 31A2 fractures showed varying levels of comminution. 31A3 fractures extended from the lesser trochanter distally (Figure 1). Trochanteric fracture classification was described by Muller et al. [7].

**Figure 2:**
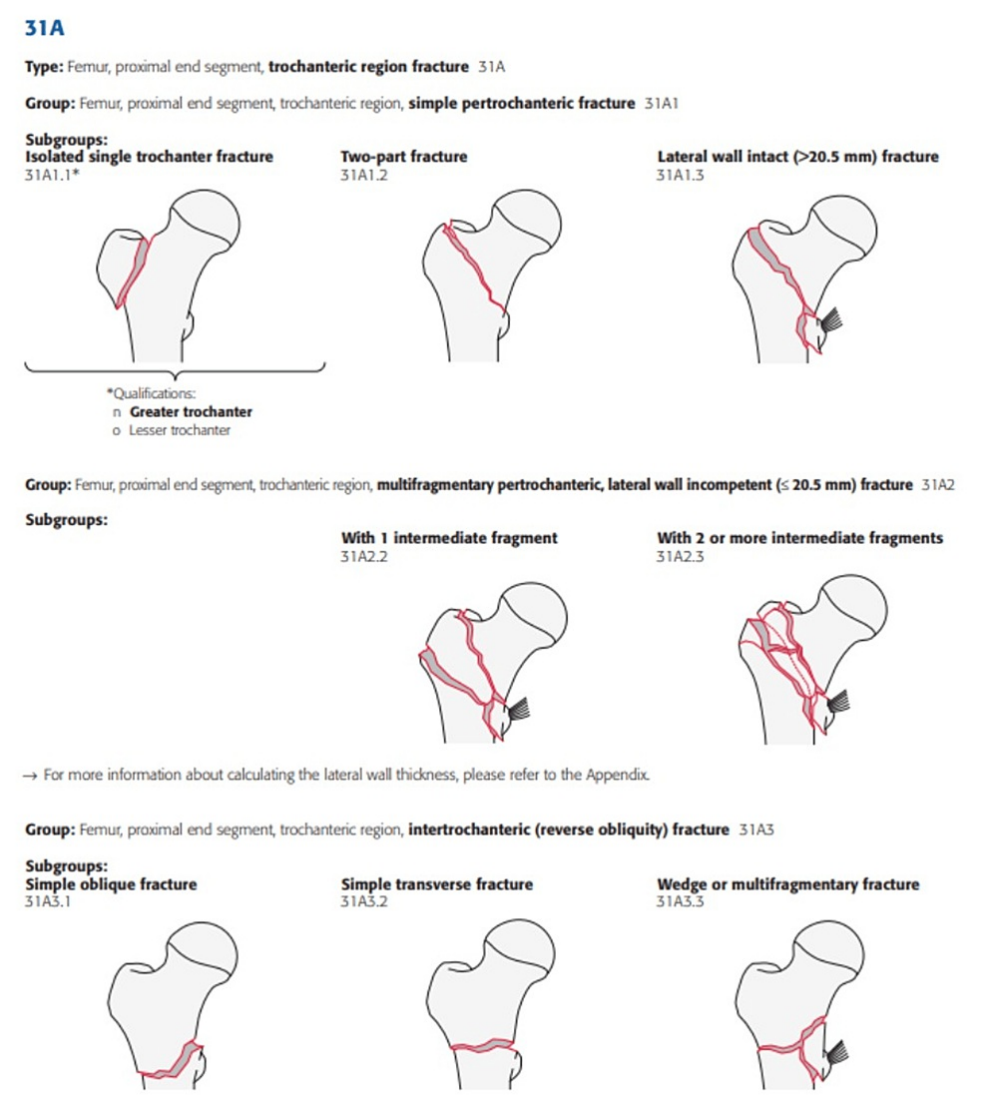
The 2018 AO/OTA TF classification is based on fracture orientation, comminution and involvement of the lateral wall. The 31A1.1 fractures are isolated fractures of the greater or lesser trochanter, while 31A1.2 and 1.3 fractures are simple pertrochanteric fractures with a competent lateral wall, i.e., lateral wall thickness >20.5 mm. Fractures classified as 31A2 have an incompetent lateral wall (lateral wall thickness <20.5 mm) with varying levels of fracture comminution. The 31A3 fractures consist of fractures running from the lesser trochanter inferiorly to the lateral femoral cortex in an oblique or transverse orientation, with or without comminution. TF: trochanteric fracture. Source: Reproduced from Meinberg et al. [6] with permission from the Journal of Orthopaedic Trauma.

In 2018, AO/OTA updated the classification of TFs [6]. According to the updated classification, the integrity of the lateral wall determined the stability of TF [6,9,10]. This classification was also divided into three broad groups: 31A1 fractures constituted simple pertrochanteric fractures with a minimum lateral wall thickness of 20.5 mm measured from a point 30 mm distal to the innominate tubercle at a 135° angle to the fracture site. The 31A2 fractures had an incompetent lateral wall, i.e., lateral wall thickness less than 20.5 mm, and 31A3 was as described in the 1990 classification (Figure 2) [6].

The NICE guidelines for the management of TF were updated due to the change in AO/OTA classification of TF in 2018 [5,6]. However, the advice for management of TF before and after the update remained unchanged and recommended SHS over IMN for fractures above and including the lesser trochanter [5].

Compliant surgeries constitute 88.33% and 89.13% before and after the update, respectively. Our study suggests that decision-making and management prior to and after the update have remained unchanged. In the United Kingdom, compliance from October 2022 to March 2023 was about 67% [11]. Across both groups, compliance with management of 31A1 fractures was good (95% Group A and 100% Group B). There was a tendency for 31A2 fractures to be non-compliant with guidelines, and the compliance rate was 76.92% (n=20) and 82.35% (n=14) in Group A and Group B, respectively. While all 31A3 fractures were compliant in Group A, the compliance in Group B was 84.62% (n=11).

We propose that non-compliance with guidelines is multifactorial. Variation in surgical management of fractures could be due to bias as a result of surgeon preferences and proficiency in one procedure over the other. Multiple studies have found only moderate intraobserver and interobserver variability with the 2018 AO/OTA classification [12,13]. There is a possibility that the fractures were wrongly classified by the operating surgeon, and hence patients received a surgical procedure that was non-compliant. Additionally, there may be a lack of awareness among surgeons about the guidelines. While it may be difficult to change preferences and bias, a lack of awareness could be addressed by educating surgeons about the guidelines, which state that as per current evidence both extramedullary and intramedullary implants have equivalent outcomes for extracapsular fracture above and including the lesser trochanter. A systematic review of evidence by NICE showed no statistically significant difference between SHS and IMN in mortality, reoperation rate, postoperative mobility score, implant cut-out, infection, non-union, length of hospital stay and pain score for 31A1 and A2 fractures [14]. A Cochrane review of 43 randomised and quasi-randomised trials by Parker et al. [15] also found that SHS had a lower complication rate compared to IMN. In addition, IMN did not have better functional outcomes, and hence SHS was found to be a superior implant [15].

In 2010, the cost of a SHS was £252.51, short IMN £760.08 and long IMN £1175.40 [14]. Since both extramedullary and intramedullary implants have equivalent outcome measures, SHS are recommended due to their lower cost without adverse consequences for patients. Education must also aim to address the cost savings associated with using SHS over IMN, with a focus on a sustainable health economic model [14].

We acknowledge that there are a few limitations to our study. Firstly, the study would have greater significance if the sample size was large. Since the guidelines were only changed in January 2023 and the study was performed in April 2023, we collected data for a period of three months in both groups, and hence the sample size was small. A multicentre study with a large cohort would be an appropriate reflection of current surgical practice. Furthermore, while evidence states that intra- and interobserver variability could have resulted in incorrect fracture classification and, consequently, non-compliance with guidelines, we did not assess intra- and interobserver variability in our study. Lastly, while SHS and IMN are reported to have equivalent outcomes for 31A1 and A2 fractures, we did not review outcomes in our study. Reviewing outcomes would have given our study greater validity.

## Conclusions

Our study suggests that district general hospitals are capable of achieving high rates of compliance with the updated NICE guidelines for the management of TFs. 31A2 fractures have a higher tendency to be non-compliant with guidelines. Causes for non-compliance with NICE guidelines could be due to surgeon preference and proficiency in a particular procedure, erroneous fracture classification and a lack of awareness of the updated guidelines. Education about guidelines will help improve compliance with NICE guidelines and ensure cost-effective treatment.
